# A giant low-grade appendiceal mucinous neoplasm (LAMN) presenting as ileocecal intussusception: a case report

**DOI:** 10.1093/jscr/rjad273

**Published:** 2023-05-27

**Authors:** Stavros C Liapis, Konstantinos Perivoliotis, Kyriakos Psarianos, Charito Chatzinikolaou, Amalia I Moula, Pavlos Skoufogiannis, Ioannis Balogiannis, Dimitrios Lytras

**Affiliations:** Department of Surgery, General Hospital of Volos, Magnesia, Greece; Department of Surgery, General Hospital of Volos, Magnesia, Greece; Department of Surgery, General Hospital of Volos, Magnesia, Greece; Department of Surgery, General Hospital of Volos, Magnesia, Greece; Department of Surgery, General Hospital of Volos, Magnesia, Greece; Department of Pathology, General Hospital of Volos, Magnesia, Greece; Department of Surgery, General University Hospital of Larissa, Thessaly, Greece; Department of Surgery, General Hospital of Volos, Magnesia, Greece

**Keywords:** low-grade, appendiceal, mucinous, neoplasm, mucocele, intussusception

## Abstract

We report the rare case of a giant low-grade appendiceal mucinous neoplasm (LAMN), presenting as ileocecal intussusception. An 80-year-old woman presented in the emergency department of our institution with progressively worsening diffuse abdominal pain during the last 24 h. A CT scan revealed a giant abdominal mass (98.7 × 127.3 × 107.6 mm) with air-fluid level and imaging characteristics of ileocecal intussusception. An emergency exploratory laparotomy was performed, and a well-circumscribed cystic mass deriving from the appendix was found. A right hemicolectomy was performed, and the histopathological examination confirmed the diagnosis of LAMN. This report aims to raise awareness among surgeons and radiologists, about LAMNs as a differential diagnosis of right iliac fossa masses presenting as acute abdomen.

## INTRODUCTION

Appendiceal neoplasms represent 0.5% of all gastrointestinal tract tumors, whereas almost 0.9–1.7% of appendectomy specimens may harbor malignancy [1]. Most appendiceal masses consist of primary epithelial neoplasms and neuroendocrine tumors, with epithelial tumors being the most common. The latter are further classified into mucinous and non-mucinous subtypes [2].

Appendiceal mucinous neoplasms (AMNs) are rare tumors, with a benign to malignant potential [3]. AMNs are classified as high-grade AMNs, mucinous adenocarcinomas, poorly differentiated mucinous adenocarcinomas with signet ring features and low-grade appendiceal mucinous neoplasms (LAMNs) [4, 5, 6]. Most LAMNs are incidental findings during an appendicectomy; weight loss, nausea and vomiting, palpable mass, and abdominal distention have been described as associated clinical manifestations [7, 8]. However, to the best of our knowledge, giant mucocele mimicking ileocecal intussusception has been rarely reported.

Herein, we present a rare case of a giant LAMN presenting as ileocecal intussusception.

## CASE REPORT

An 80-year-old postmenopausal woman presented to the emergency department of our institution with progressively worsening diffuse abdominal pain for the past 24 h. The patient also reported several episodes of vomiting. Her medical history included two cesarean deliveries, arterial hypertension, dyslipidemia, trigeminal neuralgia and a reducible umbilical hernia. There was no report of previous endoscopic or imaging studies of the abdomen. She was neither a smoker nor an alcohol consumer. Her BMI was 25 kg/m^2^.

The patient presented hemodynamically stable, with clinically generalized peritonism. Abdominal examination revealed diffuse tenderness, guarding and a positive Blumberg’s sign. Abdominal auscultation confirmed the absence of bowel sounds. Digital rectal examination showed an empty rectum. The following vital signs were recorded: blood pressure: 110/70 mmHg, pulse rate: 98 beats/min, SpO2: 96%, body temperature: 37.1°C.

Laboratory examinations (Table 1) revealed leukocytosis (11.60 K/μl) and an increased C-reactive protein (CRP; 308.70 mg/L).

**Table 1:** Laboratory tests.

**Examination**	**Value**	**Normal range**
Hemoglobin (HGB)	11.9 gr/dl	11.50–15.50
Hematocrit (HCT)	36.7%	35.0–47.0
White blood cell (WBC)	11.60 K/μl	3.50–11.00
Neutrophils (NEUT)	8.82 K/ml (76%)	2.00–7.00
Lymphocytes (LYMP)	1.23 K/ml (10.6%)	1.50–4.00
NLR ratio^a^	7.17	NLR-L (<3), NLR-H (>3)
Platelets (PLT)	243 K/μl	150.0–400.0
PLR ratio^b^	0.19	PLR-L (<300), PLR-H (>300)
CRP	308.70 mg/L	0–5 mg/L
Glucose	109 mg/dl	70–115
Urea	33 mg/dl	10–55
Creatinine	0.6 mg/dl	0.9–1.4
SGOT, AST	23 IU/l	5–40
SGPT, ALT	18 IU/l	5–40
Alkaline phosphatase (ALP)	101 IU/l	30–120
Gamma-glutamyl transferase (GGT)	72 IU/l	10–50
Total bilirubin (TBIL)	1.6 mg/dl	0.3–1.2
Amylase	27 IU/l	0–90
Prothrombin time	14.2 s	11.0–12.5 s
APTT	28.2 s	25.0–40.0 s
International normalized ratio (INR)	1.08	<1.2
D-DIMER	2.61 mg/L	0.00–0.50
Sodium (Na^+^)	135 meq/L	132–152
Potassium (K^+^)	4.6 meq/L	3.2–5.2
Albumin	2.9 g/dl	3.5–5

^a^NLR-L, neutrophil-to-lymphocyte-ratio low; NLR-H, neutrophil-to-lymphocyte-ratio high.

^b^PLR-L, platelet-to-lymphocyte-ratio low; PLR-H, platelet-to-lymphocyte-ratio high.

**Figure 1:**
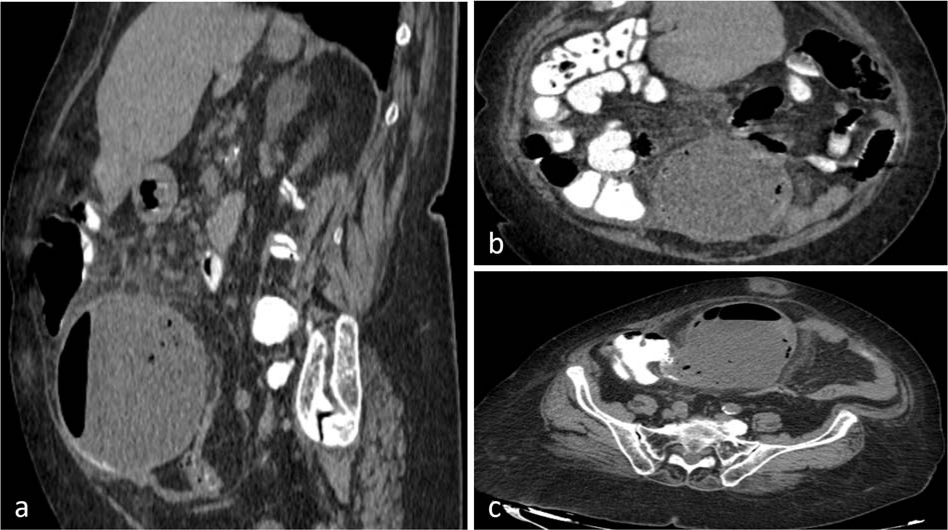
CT scan of the abdomen and pelvis with sagittal (**a**), coronal (**b**), axial (**c**) and views of a well-circumscribed, low attenuation, spherical cystic mass, with internal homogeneous non-enhancing contents and hydro-aerial levels, squalidness of surrounding fat without lymphadenopathy and slightly thickened enhancing wall with mural calcification, located anterior to the right psoas muscle contiguous with the base of the cecum.

Abdominal ultrasound revealed a dense, fluid-filled mass in the right iliac region, with a significant amount of free intraperitoneal fluid adjacent to the ascending colon. The CT scan demonstrated a large lesion (98.7 × 127.3 × 107.6 mm) with a fluid-gas level that appeared to be attached at the base of the cecum (Fig. 1a–c). It extended from central pelvis to the right lower quadrant of the abdomen with no anatomical attachment to the right ovary. No regional lymphadenopathy was noted (Fig. 1a–c). Tumor markers (CEA, CA19-9 and CA125) were normal. Based on these findings, the initial differential diagnosis included ileocecal intussusception.

**Figure 2:**
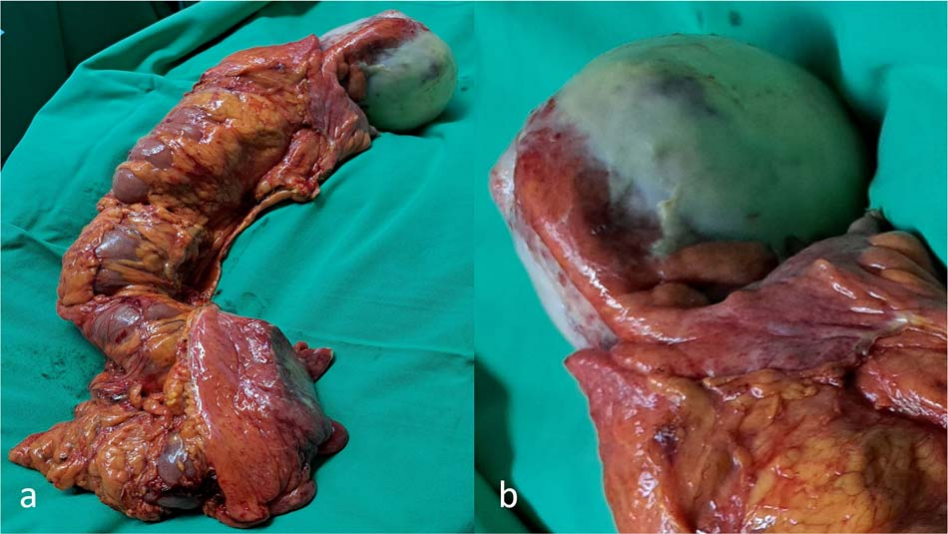
A gross appearance of the right excised colon (**a**, **b**). The circumscribed cystic mass overwhelming the appendix and extending over the cecum and extending beside the cecum, and from body to base of the appendix, without base influence (**a**, **b**).

The patient was submitted to an emergency exploratory laparotomy. Intraoperatively, a well-circumscribed cystic mass at the region of the ileo–cecal junction was found (Fig. 2a and b). The mass infiltrated the cecum and adhered to the transverse colon (Fig. 2a and b). Subsequently, a right colectomy with a stapled, side-to-side ileo-colonic anastomosis was performed. The operation was performed following the CME principles. The tumor was removed en block with the ascending colon and there was no intraoperative spillage of the cystic contents. The patient had an uneventful recovery and was discharged on postoperative Day 3.

**Figure 3:**
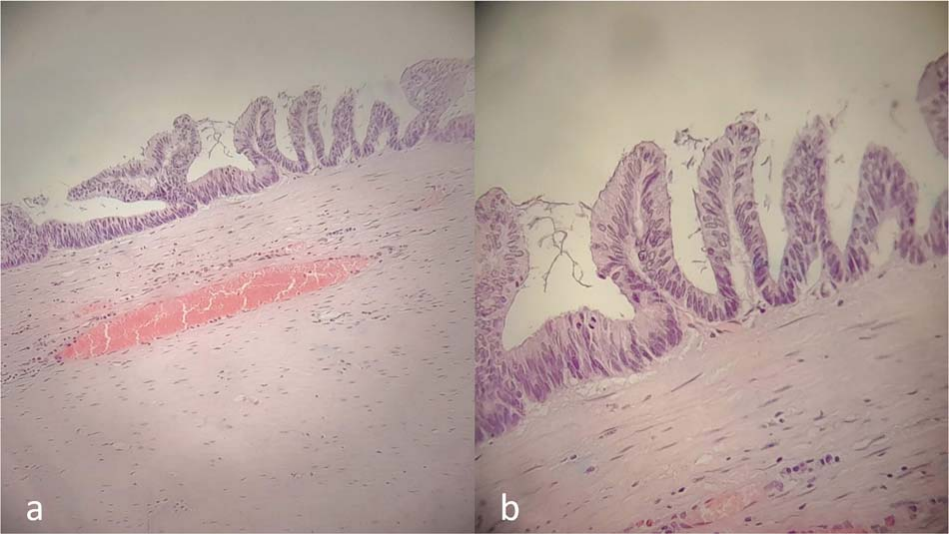
Histopathology report. Villous proliferation of mucinous epithelial cells; showing elongated nuclei and low-grade nuclear atypia (**a**, **b**).

Histology results (Fig. 3a and b) revealed a low-grade appendicular mucinous neoplasm with largest diameter of 10.5 cm, negative margins and no involved mesenteric lymph nodes. The pathological diagnosis was a LAMN with a pT3N0M0G1 (IIA) stage, using the 8th Edition of the AJCC [8]. Α tubule-villous adenoma with low-grade dysplasia was found in complete colonoscopy and was removed endoscopically. Because of the macroscopic and histopathological findings, the institutional multidisciplinary team board suggested a prophylactic CRS—HIPEC consultation. Six months later, the patient remains disease-free, with scheduled follow-up. LAMNs should be considered in the differential diagnosis of right iliac fossa masses presenting as acute abdomen.

## DISCUSSION

LAMNs are semi-malignant tumors of the appendix, diagnosed as an incidental finding in up to 1% of appendectomy specimens, at a mean age of 62.03 years, and with females being predominantly affected [9]. The term LAMNs applies to lesions without infiltrative invasion, instead of lesions with infiltrative invasion, such as ‘mucinous adenocarcinoma’ [5]. Histologically, LAMNs are characterized by low-grade cytology and any of the following: loss of the lamina propria and muscularis mucosae, fibrosis of the submucosa ‘pushing’ diverticulum-like growth into the wall, dissection of acellular mucin in the wall and mucin and/or neoplastic cells outside of the appendix [5, 6].

Preoperative diagnosis of LAMNs remains challenging, because of clinical heterogeneity. An acute appendicitis-like presentation with right lower quadrant pain is common for early stage disease, as opposed to advanced-stage symptoms like chronic abdominal pain, urinary retention, constipation and new-onset umbilical or inguinal hernias because of increased abdominal pressure [7, 8]. Ileocecal/ileocolic intussusception is an extremely rare associated condition with few reported cases [10]. This patient’s presentation was characterized by early and advanced-stage symptoms, with evidence of appendicular infection and bowel obstruction, without the accumulation of mucinous ascites in the peritoneum.

Preoperatively contrast-enhanced CT has an accuracy of 89.7% [11]. For the majority of advance-staged patients, laboratory findings are nonspecific and include anemia or elevated tumor markers [12, 13, 14]. Additionally, mucocele rupture with mucus extrusion produces an inflammatory response to the mucus, simulating ruptured appendicitis [5]. Furthermore, inflammation markers like neutrophil-to-lymphocyte-ratio (NLR) and platelet-to-lymphocyte-ratio (PLR) can be used as prognosticators for patients with cellular PMP (M1b stage) [14].

The tumor mass of the reported case was characterized by the absence of perforation, a strong inflammatory response (CRP; 308.70 mg/L) and negative tumor markers, despite the advanced presentation, disease stage and association with NLR; 7.17 (neutrophil-to-lymphocyte-ratio high, NLR-H) and PLR; 0.19 (platelet-to-lymphocyte-ratio low, PLR-L) (Table 1).

Generally, small LAMNs can be treated with open or laparoscopic appendectomy, but for larger LAMNs, laparotomy may be necessary because of possible cecal involvement [15]. If LAMN is suspected, careful removal is indicated to avoid cystic wall rupture and spillage of any potentially malignant cells or mucus that may result to PMP [13].

In our case, a giant LAMN presented as an ileocecal intussusception. Because of the intraoperative findings a CME right hemicolectomy was performed.

## CONFLICT OF INTEREST STATEMENT

None declared.
